# Women, Younger Clinicians’, and Caregivers’ Experiences of Burnout and Well-being During COVID-19 in a US Healthcare System

**DOI:** 10.1007/s11606-021-07134-4

**Published:** 2021-11-02

**Authors:** Ellis C. Dillon, Cheryl D. Stults, Sien Deng, Meghan Martinez, Nina Szwerinski, P.T. Koenig, Laurie Gregg, Jill Kacher Cobb, Elizabeth Mahler, Dominick L. Frosch, Sarina Le Sieur, Melissa Hanley, Suzanne Pertsch

**Affiliations:** 1 Center for Health Systems Research, Sutter Health and Palo Alto Medical Foundation Research Institute, 795 El Camino Real, Palo Alto, CA 94301 USA; 2 Sutter Medical Group, Sacramento, CA USA; 3 Sutter Medical Center Sacramento and Sutter Independent Physicians, Sacramento, CA USA; 4 Novato Community Hospital, Sutter Health, Novato, CA USA; 5 John Muir Health, Walnut Creek, CA USA; 6 Sutter Health, Sacramento, CA USA; 7 Palo Alto Foundation Medical Group, Palo Alto, CA USA

**Keywords:** burnout, well-being, gender, clinicians, COVID-19

## Abstract

**Background:**

The COVID-19 pandemic brought rapid changes to the work and personal lives of clinicians.

**Objective:**

To assess clinician burnout and well-being during the COVID-19 pandemic and guide healthcare system improvement efforts.

**Design:**

A survey asking about clinician burnout, well-being, and work experiences.

**Participants:**

Surveys distributed to 8141 clinicians from June to August 2020 in 9 medical groups and 17 hospitals at Sutter Health, a large healthcare system in Northern California.

**Main Measures:**

Burnout was the primary outcome, and other indicators of well-being and work experience were also measured. Descriptive statistics and multivariate logistic regression analyses were performed. All statistical inferences were based on weighted estimates adjusting for response bias.

**Key Results:**

A total of 3176 clinicians (39.0%) responded to the survey. Weighted results showed 29.2% reported burnout, and burnout was more common among women than among men (39.0% vs. 22.7%, *p*<0.01). In multivariate models, being a woman was associated with increased odds of reporting burnout (OR=2.19, 95% CI: 1.51–3.17) and being 55+ years old with lower odds (OR=0.54, 95% CI: 0.34–0.87). More women than men reported that childcare/caregiving was impacting work (32.9% vs. 19.0%, *p*<0.01). Even after controlling for age and gender, clinicians who reported childcare/caregiving responsibilities impacted their work had substantially higher odds of reporting burnout (OR=2.19, 95% CI: 1.54–3.11). Other factors associated with higher burnout included worrying about safety at work, being given additional work tasks, concern about losing one’s job, and working in emergency medicine or radiology. Protective factors included believing one’s concerns will be acted upon and feeling highly valued.

**Conclusions:**

This large survey found the pandemic disproportionally impacted women, younger clinicians, and those whose caregiving responsibilities impacted their work. These results highlight the need for a holistic and targeted strategy for improving clinician well-being that addresses the needs of women, younger clinicians, and those with caregiving responsibilities.

**Supplementary Information:**

The online version contains supplementary material available at 10.1007/s11606-021-07134-4.

## INTRODUCTION

The COVID-19 pandemic has bought renewed focus and concern to the issue of healthcare clinician burnout. Prior to the pandemic, 50% or more of clinicians were reporting burnout, higher than any other profession.^1–3^ Burnout, defined by emotional exhaustion, depersonalization, and low personal accomplishment brought on by work,^4^ is part of the larger construct of well-being, defined as “quality of life, which includes the absence of ill-being and the presence of positive physical, mental, social, and integrated well-being… across personal and work-life domains.”^5^ Factors impacting well-being include autonomy, financial stability, family dynamics and caregiving, organizational dynamics, and social support,^6–8^ all areas that have been significantly affected by the pandemic. This trend is especially concerning as previous research has shown burnout influences the quality of patient care, medical errors, patient experience, clinicians’ personal relationships, alcohol use, depression, suicidality, and early retirement.^9–13^ Furthermore, clinician burnout is directly related to the structure and organization of health care, for example, work policies, environment, workload, personal autonomy, and leadership.^8,14–16^

Research finds that both female physicians and younger physicians experience higher rates of burnout than their male and older counterparts. Two separate 2020 studies found almost 50% of women physicians reporting burnout, compared to 37% of male physicians in one study and 41.5% in the other.^2,17^ Having children at home and work-life integration are two factors commonly identified as contributing to burnout among female physicians, ^18,19^ causing women to be more likely to reduce work hours to manage caregiving responsibilities.^20–22^ Similarly, Dyrbye et al. reported that physicians younger than 55 had 200% increased risk of burnout compared to physicians older than 55,^23^ and a 2020 Medscape survey found that 48% of Generation X clinicians (40–54-year-olds) reported burnout, compared to 38% of Millennials and 39% of Baby Boomers.^24^

Not surprisingly, the stress of the COVID-19 pandemic on individual clinicians and healthcare systems may have influenced burnout. Well-being during the pandemic is influenced by job stress, fear of infection, interpersonal and interprofessional relationships, adequacy of resources, caregiving obligations, and access to rapid testing.^25,26^ A 2020 global study of intensive care unit specialists found burnout in North America in the 50–70% range, and that younger age and female sex were associated with severe burnout.^27^ The pandemic may be widening gender inequalities with challenges for working mothers’ managing a “second shift” of caregiving, household responsibilities, and “distance learning.”^28–30^ Prasad and colleagues found that women reported greater fear of exposure, prevalence of anxiety and depression, burnout, and work overload, and feeling less valued by their organization.^2^ Age also contributes to decreased well-being during the pandemic, with younger physicians reporting more depression, stress, anxiety, psychological burden, higher emotional exhaustion, and less personal accomplishment than their older counterparts.^31,32^

This study investigates the relationships between burnout and gender, age, caregiving, and holistic well-being^33^ during the COVID-19 pandemic to identify areas of possible opportunities for health system improvement.

## METHODS

Sutter Health is a large integrated healthcare organization in Northern California serving approximately 3 million patients with 22 hospitals and a network of medical foundations. A team of researchers and clinical and operational well-being leaders planned a COVID-19 pulse well-being survey aligned with Shanafelt et al.’s framework for healthcare professionals during COVID-19: “hear me, protect me, prepare me, support me, and care for me.”^26^ The survey goals were to provide an opportunity to express appreciation by listening to clinicians about their experiences, and gather meaningful and actionable information to inform leadership about opportunities for improvement.

### Survey Development and Distribution

The team developed a short, confidential survey covering 5 key domains: overall burnout (1 item),^34^ leadership and recognition (4 items), the impact of COVID-19 on work and life (6 items), support desired by clinicians (6 items), and how work responsibilities changed during the pandemic (5 items) (see Appendix). One medical group opted out of asking about burnout.

Clinicians from 9 medical groups and 17 Sutter hospitals were surveyed, including rural and urban counties in the Bay Area and Central Valley. Administrators provided lists of clinicians (physicians and advance practice clinicians) which ranged in size from 99 to 1748 for medical groups and from 41 to 1333 for hospitals. Both medical group and hospital clinicians included individuals who worked in ambulatory care, inpatient care, and both.

Between June and August 2020, 8141 clinicians received a survey invitation via email from the REDCap survey system. Clinicians working in medical groups and hospitals (or in multiple hospitals) received multiple invitations to the survey. For these clinicians, only their first completed survey was analyzed.

### Study Outcomes and Measures

The primary outcome was *burnout* : “Overall, based on your definition of burnout, how would you rate your level of burnout?”^34^ This single-item burnout measure has been widely used and validated against the Maslach Burnout Inventory.^2,35^ Burnout was defined as answering 3, 4, or 5 (i.e., “definitely burning out…,” “symptoms of burnout won’t go away,” or “I feel completely burned out…”).

Other outcomes of interest included a question about job stress from the Mini-Z survey,^36^ and novel questions generated by the research team, e.g., decreased overall well-being: “During this time of the COVID-19 pandemic: My overall well-being has been negatively affected,” and the impact of caregiving on work: “My childcare or caregiving responsibilities are impacting my work.” These measures and questions about leadership, recognition, and impact of COVID-19 on work and life used the same five response options (strongly disagree, disagree, neutral, agree, strongly agree) with “not applicable” included for some questions. Responses were dichotomized to capture agreement: combining “agree” and “strongly agree.” One open-ended question was asked to gather more in-depth information on desired support: “Please tell us more about what can be done to better support you right now.” The rationale for these questions was to capture easily quantifiable data as well as more detailed stories and nuanced thoughts from the resulting qualitative data.

### Statistical Analyses

Descriptive statistics were used to compare clinician demographics for survey respondents versus non-respondents, with and without adjusting for response bias. Weighted bivariate analyses were conducted to compare group differences in primary measures, where chi-square statistics were used with *p* ≤ .05 as the level of significance. Two sets of weighted logistic regression models were conducted and odds ratios and 95% confidence intervals are reported. Model 1 included clinicians’ demographics (i.e., age, gender, job role, and specialty) to identify characteristics associated with burnout. Model 2 built upon Model 1 by adding responses to the other survey questions (see questions listed in Table 4) to investigate how work experiences and caregiving responsibility were associated with burnout. Model 2 was also run separately for each gender and age group to identify any variation. Inferential statistics accounted for response bias using the inverse propensity weight method.^37,38^ The propensity was estimated by a logistic regression model, where the dependent variable was survey response (yes/no) and predictors included age, gender, primary specialty, and job role (physician vs. non-physician). All analyses were completed using R version 4.0.2 and all results reported are weighted unless otherwise noted.

### Qualitative Analysis

The team conducted a thematic analysis of written comments about desired support. The qualitative analysis team reviewed the data and created a preliminary set of codes to capture key themes. A team of five coders discussed and organized the codebook and coded a subset of comments achieving inter-rater reliability of kappa 0.7. Two coders independently applied the codes to each comment using Dedoose Version 8.3.45 and then calculated the frequency of themes. This project was reviewed and approved by the Sutter Health Institutional Review Board.

## RESULTS

### Overall Findings

Out of 8141 clinicians invited, 3176 (39.0%) responded to the survey and were included in the analysis. Respondents included 51.0% women, 7.5% <35 years old, 58.8% 35–54 years old, 33.2% 55+ years old, 88.8% physicians, and 11.2% non-physicians, mostly Nurse Practitioners and Physician Assistants (Table 1). The largest respondent specialty groups were Internal Medicine General 15.6%, Family Medicine 13.8%, Internal Medicine Sub-specialties 13.2%, Pediatrics 11.1%, and Surgical Specialties 9.3%. Survey respondents differed from non-respondents as well as from the overall population on most demographic characteristics including age, gender, and specialty (Table 1). Adjusting for response weights, the corrected estimates based on respondents were no longer statistically different from the overall population for all demographic variables. Response weights were applied in all analyses to adjust for response bias. Results reported below are based on weighted statistics.

**Table 1:** Characteristics of Population, Survey Non-respondents, Respondents, and Weighted Respondents

	**Overall population (*N*=8141) %**	**Non-respondents (*N*=4965) %**	**Respondents (*N*=3176) %**	**Respondents adjusted with response weight %**
**Gender**^**a,b**^				
Female	44.8	40.8	51.0	44.6
Male	54.9	58.8	48.8	55.1
Not available	0.3	0.4	0.2	0.3
**Age**^**a**^				
<35	8.3	8.9	7.4	7.6
35–54	57.6	56.8	58.8	56.3
55+	33.2	33.2	33.2	35.1
Not available	0.9	1.1	0.6	1.0
**Role**				
Physician	89.6	90.0	88.8	90.0
Non-physician	10.4	10.0	11.2	10.0
**Specialty**^**a,b**^				
Internal Medicine General	13.2	11.7	15.6	13.4
Internal Medicine Subspecialty	12.1	11.5	13.2	12.2
Surgery	10.4	11.1	9.3	10.4
Family Medicine	9.6	7.0	13.8	9.6
Pediatrics	8.9	7.5	11.1	9.0
OB-GYN	5.2	4.6	6.2	5.2
Anesthesiology	4.9	4.4	5.6	4.9
Emergency Medicine	4.1	3.1	5.7	4.1
Radiology	3.8	3.9	3.6	3.8
Urgent care/walk-in care	3.1	2.9	3.3	3.2
Psychiatry	2.2	2.4	1.8	2.2
Ophthalmology	2.1	2.2	1.9	2.0
Dermatology	1.5	1.1	2.0	1.4
Neurology	1.4	1.2	1.8	1.5
Other ^c^	3.3	2.8	3.9	3.3
Not available	14.2	22.6	1.2	13.8

No statistical difference between adjusted response and all sample (*p*≥0.05)

^a^Variables that are statistically different between response and non-response (*p*<0.05)

^b^Variables that are statistically different between response and all sample (*p*<0.05)

^c^“Other” specialty includes Otolaryngology, Pathology, Physical Medicine And Rehabilitation, Radiation Oncology, and other specialties that are less than 100 participants. For survey respondents who did not report specialty and for clinicians who did not respond to the survey (non-responders), specialty area was retrieved from electronic health record data. Similar clinical sub-specialties were grouped together

**Fig. 1:**
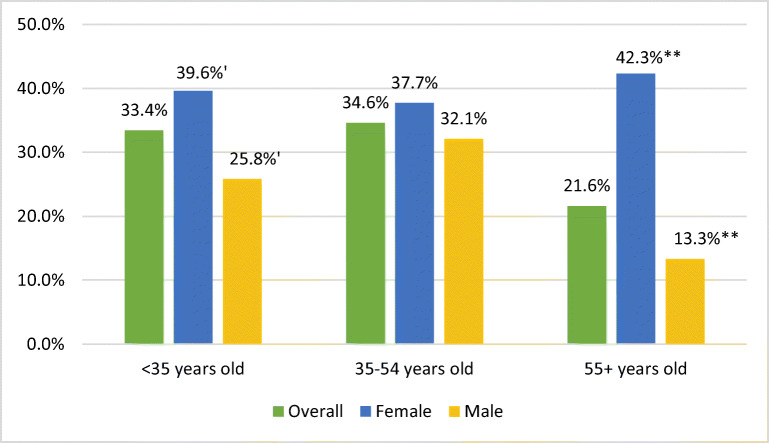
Percentage of clinicians reporting burnout by gender within each age group. ‘*p*<0.10, **p<0.01.

Overall, 29.2% of clinicians reported burnout. Compared to men, more women reported burnout (39.0% vs 22.7%, *p*<0.0001). Rates of burnout also varied by age, with more clinicians under 35 years old (33.4%) and 35–54 years old (34.6%) reporting burnout compared to 55+ years olds (21.6%) (*p*<0.0001). More 35–54-year-olds reported severe burnout (i.e., selecting 4 or 5) compared to the other age groups (12% vs. 7.4% under 35 years old and 4.6% 55+ years olds) (Table 2). The largest variation in percent of clinicians reporting burnout by gender occurred among those under 35 years old (women 39.6% vs men 25.8%) and 55+ years old (women 42.3% vs men 13.3%) (Fig. 1).

**Table 2:** Responses to Survey Questions by Respondent Gender, Age, and Role (*N*=3176)

			**Gender (weighted)**		**Age (weighted)**			**Job role (weighted)**	
	**All clinicians (unweighted)**	**All clinicians (weighted)**	**Female**	**Male**	**<35**	**35–54**	**55+**	**Non-physician**	**Physician**
	***N*(%)**	**%**	**%**	**%**	**%**	**%**	**%**	**%**	**%**
**Survey questions (selecting “agree”/“strongly agree”)**									
I feel highly valued.	2112 (68.4)	67.9	63.7**	71.5**	67.5**	64.5**	73.8**	63.7	68.4
I feel supported and listened to by my leadership.	2095 (68.1)	67.8	63.0**	71.6**	72.4	65.2	70.6	64.9	68.1
I feel comfortable providing feedback or concerns to my leaders.	2268 (73.6)	72.3	69.4	74.4	81.0**	69.0**	75.4**	73.6	72.1
I believe my concerns will be acted upon.	1667 (54.1)	54.6	51.0*	57.6*	59.1	52.4	57.3	52.8	54.8
I am worried about my safety at work.	1302 (42.4)	43.9	45.6	42.5	38.3	45.7	42.0	35.2*	44.8*
My overall well-being has been negatively affected.	1448 (47.4)	46.3	51.1**	42.5**	45.3***	50.8***	39.5***	39.4*	47.1*
My childcare or caregiving responsibilities are impacting my work.	773 (25.3)	25.2	32.9***	19.0***	16.6***	37.5***	7.4***	22.5	25.6
I am concerned about loss of income.	2142 (70.0)	68.6	69.3	68.1	65.1**	72.6**	62.9**	62.8*	69.3*
I am concerned about loss of my job.	672 (22.0)	22.3	27.2***	18.3***	39.3***	23.7***	16.3***	47.4***	19.4***
I feel a great deal of stress because of my job.	1403 (45.8)	45.6	51.1***	41.3***	45.8***	51.4***	36.8***	42.7	45.9
**Overall, based on your definition of burnout, how would you rate your level of burnout? (check only one) †‡**									
***Reporting burnout (checked 3, 4, or 5)***	617 (28.9)	29.2	39.0***	22.7***	33.4***	34.6***	21.6***	28.8	29.3
1. I enjoy my work. I have no symptoms of burnout.	396 (18.5)	19.4	11.7***	24.4***	15.1***	13.8***	27.7***	17.1	19.7
2. Occasionally under stress - but I don’t feel burned out…	1123 (52.6)	51.4	49.3***	52.9***	51.5***	51.6***	50.6***	54.0	51.0
3. Definitely burning out…	465 (21.8)	20.5	30.7***	13.6***	26.0***	22.6***	17.1***	19.0	20.7
4. Symptoms of burnout won’t go away…	112 (5.2)	5.9	6.4***	5.6***	5.4***	8.0***	3.2***	7.5	5.7
5. I feel completely burned out and wonder if I can go on…	40 (1.9)	2.8	1.9***	3.5***	2.0***	4.0***	1.4***	2.4	2.9
**What can be done to better support you right now? (check all that apply)**									
More training on COVID-19.	396 (12.9)	12.6	15.1*	10.6*	21.0*	12.4*	10.9*	20.7***	11.7***
Provide more personal protective equipment (PPE).	1161 (37.5)	36.0	36.3	35.8	41.4***	39.7***	28.6***	32.1	36.5
More training on use of PPE.	357 (11.6)	11.4	12.0	10.9	12.2	10.8	12.4	10.8	11.5
Support for mental health needs.	410 (13.3)	12.4	15.6**	9.9**	17.3	13.1	10.5	17.8**	11.8**
Provide more flexibility with schedules.	839 (27.0)	25.0	29.4***	21.6***	35.8***	28.7***	16.8***	28.0	24.6
Other supports.	490 (16.5)	15.5	16.6	14.7	10.0**	14.3**	18.8**	13.1	15.8
**How have your professional responsibilities and work changed because of the COVID-19 crisis? (check all that apply)**									
I have been given additional tasks.	950 (29.9)	27.2	29.8*	25.2*	34.8***	30.6***	20.8***	32.4	26.7
The hours I work decreased.	1021 (32.1)	33.2	27.8***	37.5***	31.3**	29.4**	38.8**	36.1	32.9
The hours I work increased.	562 (17.7)	16.1	17.5	15.0	11.6*	18.3*	13.7*	6.7***	17.1***
Other changes in work.	345 (10.9)	9.3	12.6***	6.6***	11.1	8.8	9.7	12.9	8.9
No changes in work.	503 (15.8)	16.7	15.0	18.1	13.9	17.1	17.0	0.65	13.9

*p<0.05; **p<0.01; ***p<0.001. All statistically significant differences displayed in bold font

†Primary outcomes

‡Full text of burnout question: Overall, based on your definition of burnout, how would you rate your level of burnout? (check only one)

1. I enjoy my work. I have no symptoms of burnout.

2. Occasionally I am under stress, and I don’t always have as much energy as I once did, but I don’t feel burned out.

3. I am definitely burning out and have one or more symptoms of burnout, such as physical or emotional exhaustion.

4. The symptoms of burnout that I’m experiencing won’t go away. I think about frustration at work a lot.

5. I feel completely burned out and often wonder if I can go on. I am at the point where I may need some changes or may need to seek some sort of help.

Overall, 25.2% of clinicians reported caregiving responsibilities were impacting work. More women than men (32.9% vs. 19.0%, *p*<0.0001) and more middle-aged clinicians (37.5% 35–54 years old vs. 16.6% <35-year-olds and 7.4% 55+ years olds, *p*<0.0001) reported caregiving impacting work. Regarding other well-being measures, 46.3% reported “My overall well-being has been negatively affected,” 43.9% were worried about safety at work, and 45.6% indicated “I feel a great deal of stress because of my job.” Compared to men, more women reported job stress (51.1% vs. 41.3%, *p*<0.0001) and decreased overall well-being (51.1% vs 42.5%, *p*<0.01). More middle-aged clinicians reported decreased overall well-being (50.8% 35–54 years olds vs. 45.3% <35 years olds and 39.5% 55+ years olds, *p*<0.0001), and job stress (51.4% 35–54 years olds vs. 45.8% <35 years olds and 36.8% 55+ years olds, *p*<0.0001). Compared to non-physicians, more physicians reported decreased overall well-being (47.1% vs. 39.4%, *p*<0.05).

Compared to men, fewer women felt highly valued (63.7% vs. 71.5%, *p*<0.01), fewer were confident their concerns would be acted upon (51.0% vs. 57.6%, *p*<0.05), and more were worried about losing their job (27.2% vs. 18.3%, *p*<0.0001).

The most frequent requests for support were for more personal protective equipment (PPE) (36.0%), flexibility with schedules (25.0%), and support for mental health needs (12.4%). Compared to men, more women desired support for mental health (15.6% vs 9.9% male, *p*<0.01) as well as more flexible schedules (29.4% vs 21.6% male, *p*<0.0001). Younger clinicians desired more support for flexible schedules (35.8% <35-year-olds and 28.7% 35–54-year-olds vs 16.8% 55+-year-olds, *p*<0.0001).

### Factors Associated with Burnout

#### Model 1

The first weighted multivariate logistic regression only included demographic variables (i.e., age, gender, job role, and primary specialty) to investigate their associations with reporting burnout (Table 3). Controlling for other covariates, age and gender were significantly associated with burnout, with women being more likely to report burnout than men (OR=2.19, 95% CI: 1.51–3.17), and clinicians 55+ years old less likely to report burnout (OR=0.54, 95% CI: 0.34–0.87). Moreover, clinicians whose primary specialty was emergency medicine had higher odds of reporting burnout (OR=1.58, 95% CI: 1.02–2.43) as did those in radiology (OR=1.87, 95% CI: 1.1–3.18).

**Table 3:** Weighted Logistic Regression Analysis for Demographics Associated with Overall Burnout (Model 1)

	**Overall burnout**
**Variables†**	OR (95% CI)
**Age (reference = age<35))**	
35–54	0.98 (0.66–1.46)
55+	0.54** (0.34–0.87)
**Female**	2.19*** (1.51–3.17)
**Physician (reference = non-physician)**	1.39 (0.92–2.09)
**Specialty (reference = Internal Medicine-General)**	
Anesthesiology	0.93 (0.57–1.52)
Dermatology	1.46 (0.56–3.77)
Emergency Medicine	1.58* (1.02–2.43)
Family Medicine	1.18 (0.8–1.75)
Internal Medicine Subspecialty	1.06 (0.71–1.56)
Neurology	1.49 (0.71–3.1)
OB-GYN	1.09 (0.69–1.73)
Ophthalmology	1.12 (0.49–2.58)
Pediatrics	1.09 (0.7–1.68)
Psychiatry	1.99' (0.98–4.02)
Radiology	1.87* (1.1-3.18)
Surgery	0.93 (0.61–1.43)
Urgent care/express care/walk-in	0.6 (0.28–1.3)
Other	1.78 (0.86–3.7)

'*p*<0.1, *p<0.05; **p<0.01; ***p<0.001. *OR* odds ratio, *95% CI* 95% confidence interval.

†Model 1 only included the variables listed in the table above

All statistically significant differences displayed in bold font

#### Model 2

In Model 2, measures of well-being and work experience were included with demographic covariates from Model 1 to explore associations with burnout. Model 2 was also run separately for each age group and each gender group to identify any relationship variation (Table 4). After controlling for demographic covariates, Model 2 for all respondents showed that those reporting caregiving responsibilities impacting work (OR=2.19, 95% CI: 1.54–3.11), being concerned about loss of job (OR=2.19, 95% CI: 1.42–3.37), being worried about work safety (OR=1.67, 95% CI: 1.23–2.26), and being given additional tasks (OR=1.79, 95% CI: 1.33–2.42) were more likely to report burnout. On the other hand, clinicians who felt highly valued (OR=0.45, 95% CI: 0.3–0.68) and who felt their concerns would be acted upon (OR=0.57 95% CI: 0.4–0.81) had lower odds of reporting burnout.

**Table 4:** Weighted Logistic Regression Analyses for Factors Associated with Overall Burnout (Model 2)

	**Overall**^**a**^	**Age Groups**^**b**^			**Gender groups**^**c**^	
	**All respondents**	**<35**	**35–54**	**55+**	**Female**	**Male**
	OR (95% CI)	OR (95% CI)	OR (95% CI)	OR (95% CI)	OR (95% CI)	OR (95% CI)
**Dichotomous survey questions response (agree vs. disagree, reference = disagree)**						
I feel highly valued.	0.45***(0.3–0.68)	0.21**(0.07–0.64)	0.47**(0.28–0.8)	0.63(0.32–1.23)	0.63*(0.41–0.96)	0.33***(0.18–0.58)
I feel supported and listened to by my leadership.	0.76(0.47–1.22)	0.44(0.15–1.28)	0.83(0.43–1.58)	0.55(0.29–1.05)	0.66'(0.4–1.07)	0.87(0.43–1.78)
I feel comfortable providing feedback or concerns to my leaders.	0.94(0.61–1.46)	2.81(0.78–10.15)	0.8(0.44–1.49)	1.18(0.61–2.29)	1.26(0.77–2.08)	0.75(0.4–1.41)
I believe my concerns will be acted upon.	0.57**(0.4–0.81)	0.59(0.21–1.69)	0.52**(0.33–0.8)	0.78(0.44–1.4)	0.67'(0.44–1.04)	0.5**(0.3–0.84)
I am worried about my safety at work.	1.67**(1.23–2.26)	1.49(0.57–3.88)	1.71**(1.21–2.42)	1.44(0.84–2.46)	1.47'(0.99–2.2)	1.84**(1.27–2.67)
My childcare or caregiving responsibilities are impacting my work.	2.19***(1.54–3.11)	1.35(0.47–3.89)	2.47***(1.68–3.64)	2.05*(1.05–4)	2.2***(1.5–3.24)	2.44***(1.49–3.99)
I am concerned about loss of income.	1.12(0.8–1.58)	1.02(0.39–2.71)	1.06(0.7–1.6)	1.18(0.68–2.05)	0.86(0.56–1.31)	1.6'(1–2.55)
I am concerned about loss of my job.	2.19***(1.42–3.37)	0.46(0.17–1.23)	2.21**(1.38–3.53)	3.21**(1.57–6.55)	2.47**(1.48–4.14)	1.51(0.91–2.49)
**Dichotomous survey questions response (yes vs. no, reference = no)**						
I have been given additional tasks.	1.79***(1.33–2.42)	2.92*(1.09–7.83)	1.83**(1.25–2.69)	1.39(0.81–2.39)	2.26***(1.53–3.33)	1.42(0.91–2.19)
The hours I work decreased.	0.75(0.52–1.07)	0.71(0.26–1.91)	0.83(0.55–1.25)	0.62'(0.35–1.09)	0.85(0.54–1.35)	0.72(0.47–1.09)
The hours I work increased.	1.38(0.93–2.04)	1.25(0.34–4.53)	1.27(0.79–2.06)	1.27(0.63–2.55)	1.75*(1.05–2.91)	1.08(0.61–1.89)
Other changes in work.	0.76(0.47–1.24)	2.00(0.47–8.46)	0.87(0.53–1.43)	0.47(0.16–1.36)	0.87(0.49–1.53)	0.62(0.29–1.33)

'*p*<0.1; *p<0.05; **p<0.01; ***p<0.001. *OR* odds ratio, *95% CI* 95% confidence interval. All statistically significant differences displayed in bold font.

^a^Model 2 for all respondents adjusted for covariates: age, gender, job role, and specialty

^b^Model 2 for each age group adjusted for covariates: gender and job role

^c^Model 2 for each gender group adjusted for covariates: age and job role

Subgroup analyses illustrated some differences across groups. Caregiving responsibilities impacting work and concern about losing jobs were significantly associated with burnout for 35–54- and 55+-year-old clinicians but not for clinicians under 35 years old. Feeling highly valued and being given additional tasks were significantly associated with burnout for younger and middle-age groups but not for 55+ years olds. Caregiving responsibilities impacting work and feeling highly valued were significantly associated with burnout for both female and male groups, but the relationships of burnout with concern about loss of jobs, given additional task, and increase of work hours were only significant for female clinicians.

### Clinician Comments on Open-Ended Support Question

Clinicians wrote 1726 comments responding to “Please tell us more about what can be done to better support you right now.” The top three themes were (1)*PPE or other equipment/facility needs* (*n*=402, 23.3%): “We need more protective equipment, disinfecting wipes, masks that work, N95s, gowns/labcoat-style tops… This is a huge contributor to my unhappiness” and “We need to provide all physicians appropriate work from home equipment or we will be seeing a lot of work-related injuries”; (2)*Improved communication with leadership* (*n*=363, 21.0%): “I would like leadership to visit and talk to departments regularly. Once a week on a rotational visit, or something. Drive. Make it personal. Make it worth our connection”; and (3)*Improved compensation*, e.g., requests for hazard pay, increased primary care compensation (*n*=273, 15.8%): “It truly does not feel that there has been any effort to honor the frontline workers in this regard- words are one thing, but no clear additional financial compensation/hazard pay/etc.” (Table 5).

**Table 5:** Qualitative Analysis of Response to Question “Please Tell Us More About What Can Be Done to Better Support You Right Now”

	**Overall (*N*=1726)**
**Clinicians commented they wanted…**	***N*(%)**
Personal protective equipment (PPE) or other equipment/facility needs	402 (23.3)
Communication with leadership	363 (21.0)
Compensation	273 (15.8)
Working relationships	259 (15.0)
Positive comments	230 (13.3)
COVID-19 specific training/guidelines	192 (11.1)
Non-physician, ancillary, and support staff issues	169 (9.8)
COVID-19 screening	158 (9.2)
In-personvisits/On-site safety issues	148 (8.6)
De-escalation/re-escalation plans	128 (7.4)
Flexibility/Autonomy with schedule	121 (7.0)
Video visit issues	117 (6.8)
Recognition/Appreciation/Positive reinforcement	77 (4.5)
Work-life balance	35 (2.0)
**Total comments**	1726 (100)

A clinician comment may include more than one theme. *N*=1726 unique comments

## DISCUSSION

This survey measured different dimensions to comprehensively assess clinician burnout, well-being, and work experiences. Among clinicians surveyed, 29% reported burnout, 46% reported decreased overall well-being, and 46% felt a great deal of job stress, providing support that the impact of the pandemic went beyond burnout. While fewer clinicians in this survey reported burnou﻿t, the large number reporting decreased well-being highlights that measuring well-being requires a more holistic and encompassing measure than burnout alone.^33^ Engagement and fulfillment at work are also important considerations in healthcare worker well-being.^39,40^ Fewer clinicians in this survey reported burnout, 29%, compared to 49% in a larger national survey using the same burnout measure among various healthcare workers.^2^ It was unsurprising that Emergency Medicine clinicians were more likely to report burnout during the pandemic, but surprising that clinicians in radiology also had higher burnout, perhaps because their work or income may have been dramatically reduced by the pandemic response.

As found elsewhere,^2^ this analysis found greater challenges for women and younger workers. Women were 2.19 times as likely to report burnout and more frequently reported job stress, caregiving impacting work, and feeling less valued and heard by leadership. Clinicians under 55 years old were more likely to report burnout, job stress, decreased well-being, caregiving impacting work, concern about job loss, and feeling less valued. Interestingly, statistically significant gender differences in burnout were found among 55+-year-old clinicians (13.3% men vs. 42.3% women). While many older clinicians may have fewer caregiving responsibilities and more financial security, some may be part of a “sandwich generation” simultaneously managing caregiving responsibilities for their children and parents.^41^ The relationship between age and burnout may also be influenced by other life cycle^42^ and age cohort effects.^43^ Protective factors included believing one’s concerns will be acted upon and feeling highly valued.

Even after controlling for gender and age, caregiving was observed to have a substantial association with burnout. Caregiving impacted work for 25% of clinicians and these clinicians were 2.19 times as likely to report burnout. Further opportunities for healthcare systems to support their workforce include improving flexibility of schedules (desired by 25% of respondents), considering caregiving needs when scheduling work and meetings, enhancing time off and flexible family leave benefits, and supporting clinicians with ongoing and emergency child care or elder care resources. While the need for these changes may be more acute during the pandemic, these concerns were common before the pandemic^44^ and will continue to be of importance to an increasingly female workforce. Measuring caregiving is itself critically important as its effect persists even when controlling for age and gender.

Organizational factors are recognized as the largest drivers of burnout and well-being at work.^9,45^ As found elsewhere, this analysis found feeling highly valued lowered the odds of burnout,^2^ as did belief that one’s concerns would be acted upon, suggesting one mechanism by which leadership may influence burnout. This survey’s findings were used to prioritize and guide clinician well-being strategies, e.g., advocating for more PPE, mental health resources, leadership development events, virtual well-being forums, flexible scheduling, and focusing on gender equity.

### Limitations

This survey was limited to one healthcare system in Northern California. The population, COVID-19 transmission rates, and other factors may differ from those in other geographies or other healthcare systems. The survey was limited to physicians and advance practice clinicians whose experience may differ from the entire healthcare workforce. We used a measure of burnout that may over-report burnout,^33^ but is widely used, non-proprietary, and shorter than the 22-item MBI. This research lacked data on clinician race and ethnicity, and future research is needed to explore the intersection of race, ethnicity, age, and gender. Additionally, the 39% response rate is subject to potential unobserved bias. The timing of this survey during the summer of 2020 likely influenced concerns about safety, PPE supply, and lost income. Some of these concerns may have waned, but they offer important lessons about preparing for future pandemics. More longitudinal data are needed to understand how burnout and well-being changed before, during, and after the pandemic.

## CONCLUSION

This survey of clinicians in Northern California found the pandemic disproportionally impacted women, younger clinicians, and clinicians with caregiving responsibilities impacting their work. These groups more frequently reported burnout, decreased overall well-being, and job stress. These results highlight the need for a holistic and targeted strategy for improving clinician well-being that addresses the strategic needs of different groups and incorporates holistic understanding of their personal and professional needs during the pandemic and beyond.

## Supplementary Information

ESM 1 (DOCX 32 kb)
