# Supplementary material for: Women, Younger Clinicians’, and Caregivers’ Experiences of Burnout and Well-being During COVID-19 in a US Healthcare System
Source: J Gen Intern Med. 2021 Nov 2;37(1):145–53. doi: 10.1007/s11606-021-07134-4 (PMC8562379; doi:10.1007/s11606-021-07134-4)
Supplement: Supplementary file 1 — (DOCX 32 kb) [file 11606_2021_7134_MOESM1_ESM.docx]

# Appendix 1. Questionnaires

***Medical Group Version -* COVID-19 Joy of Work Pulse Survey**

1. **During this time of the COVID-19 pandemic:**

|  | **Strongly Disagree** | **Disagree** | **Neutral** | **Agree** | **Strongly Agree** |
| --- | --- | --- | --- | --- | --- |
| I feel highly valued. |  |  |  |  |  |
| I feel supported and listened to by my leadership. |  |  |  |  |  |
| I feel comfortable providing feedback or concerns to my leaders. |  |  |  |  |  |
| I believe my concerns will be acted upon. |  |  |  |  |  |

1. **During this time of the COVID-19 pandemic:**

|  | **Strongly Disagree** | **Disagree** | **Neutral** | **Agree** | **Strongly Agree** | **Not Applicable** |
| --- | --- | --- | --- | --- | --- | --- |
| I am worried about my safety at work. |  |  |  |  |  |  |
| My overall well-being has been negatively affected. |  |  |  |  |  |  |
| My childcare or caregiving responsibilities are impacting my work. |  |  |  |  |  |  |
| I am concerned about loss of income. |  |  |  |  |  |  |
| I am concerned about loss of my job. |  |  |  |  |  |  |
| I feel a great deal of stress because of my job. |  |  |  |  |  |  |

1. **What can be done to better support you right now? (check all that apply)**

- More training on COVID-19
- Provide more personal protective equipment (PPE)
- More training on use of PPE
- More training on telemedicine/virtual patient visits
- Additional support for remote access
- Training on video conferencing
- Support for mental health needs
- Provide more flexibility with schedules
- Other: (please specify) ________________________

1. **Please tell us more about what can be done to better support you right now.**

(write in response up to 250 words) : ________________________________

Understanding burnout remains an organizational priority, even more so during these difficult times. Please take a moment to reflect on how you are feeling overall about your work.

1. **Overall, based on your definition of burnout, how would you rate your level of burnout? (Check only one)**
   - I enjoy my work. I have no symptoms of burnout.
   - Occasionally I am under stress, and I don’t always have as much energy as I once did, but I don’t feel burned out.
   - I am definitely burning out and have one or more symptoms of burnout, such as physical or emotional exhaustion.
   - The symptoms of burnout that I’m experiencing won’t go away. I think about frustration at work a lot.
   - I feel completely burned out and often wonder if I can go on. I am at the point where I may need some changes or may need to seek some sort of help.

Please help us to help you by giving us some background on your role during the COVID-19 pandemic.

1. **How have your professional responsibilities and work changed because of the COVID-19 crisis? (check all that apply)**

- I changed from mostly in-person patient interactions to mostly virtual patient interactions
- I have been given additional tasks
- I was re-assigned to a new team or department
- The hours I work decreased
- The hours I work increased
- I began a new leadership role
- Nothing changed
- Other: (please specify) ________________________

1. **What is your home department?**

- Administration
- Allergy/Immunology
- Anesthesiology
- Anticoagulation
- Audiology
- Back Pain
- Cardiology
- Cardiothoracic Surgery
- Dermatology
- Ear Nose And Throat/Otolaryngology
- Endocrinology/Metabolism
- Express Care
- Extended Hours
- Family Medicine
- Gastroenterology
- General Surgery
- Geriatrics
- Gynecological Oncology
- Hospitalist
- Hospitalist Pediatric
- Infectious Diseases
- Integrative Medicine
- Internal Medicine
- Maternal-Fetal Medicine / Perinatology
- Medical Oncology Or Hematology/Oncology
- Neonatal Medicine
- Nephrology
- Neurological Surgery
- Neurology
- Nuclear Medicine
- Nutrition
- Obstetrics/Gynecology
- Occupational Medicine/Health
- Ophthalmology
- Optometry
- Orthopedic Surgery
- Orthopedics
- Pain Medicine
- Pathology
- Pediatric Specialty
- Pediatrics
- Physical Medicine and Rehab
- Plastic Surgery
- Podiatry
- Psychiatry/Psychology
- Pulmonology
- Radiation Oncology
- Radiation Therapy
- Radiology
- Radiology Diagnostic
- Radiology Interventional
- Reproductive Endocrinology & Infertility
- Rheumatology
- Sleep Lab
- Sports Medicine
- Surgical Oncology
- Urgent Care - Adult
- Urgent Care - Pediatrics
- Urology
- Vascular Surgery
- Walk-In Clinic
- Other: ____________________

1. **In what setting(s) have you worked during the COVID-19 pandemic? (check all that apply)**

- Outpatient - Please select all work settings where you have worked (check all that apply)
  - Telemedicine/virtual patient visits
  - In-person patient visits/patient care
  - Respiratory clinic
  - Urgent Care
  - Walk-in Care
  - Administrative/leadership work
  - Other: (please specify)___________________________
- Inpatient - Please select all departments where you have worked (check all that apply)
  - Emergency department
  - Intensive Care Unit (ICU)
  - Labor and Delivery
  - Medical-Surgical
  - Other: (please specify)___________________________
- Other work setting (please specify):___________________________

1. **Which of the following describes your role? (check all that apply)**

- Physician
- Advanced Practice Clinician (APC)
- Leadership/management role
- Other: (please specify) _________________________________

**[Language/contact info below was customized for each medical group.]**

**Thank you very much for completing this survey.**

We also encourage employees to seek support when experiencing challenges that are affecting them, a colleague, or family member. It all starts with a conversation:

Please see our [Employee Resource Guide](https://spark.adobe.com/page/AYsriULqllbIr/) with links to news and resources related to COVID-19.

• **Sutter Health’s Employee Assistance Program (EAP)**—provides free, confidential assistance, including referrals to licensed counseling professionals. Call 1-800-477-2258 or go online to [sutterhealth.org/eap](https://www.sutterhealth.org/for-employees/employee-assistance-program).

• **Physician Support Line**—a national, free, and confidential support line service made up of 600+ volunteer psychiatrists, joined together in the determined hope to provide peer support for our physician colleagues as we all navigate the COVID-19 pandemic. Call 1-888-409-0141.

• **National Suicide Prevention Lifeline**—if you are concerned for yourself or someone else, call 1-800-273-8255.

• **Crisis Text Line**—connect by text with a trained crisis counselor by texting 741741.

***Hospital Version -* COVID-19 Joy of Work Pulse Survey**

1. **What is your specialty?**
   - [customized list for each hospital inserted here]
   - Other: __________
2. **In what acute care settings have you worked during the COVID-19 pandemic?** **(check all**

**that apply)**

- - eICU
  - Emergency Department
  - Intensive Care
  - Labor and Delivery / OB-GYN
  - Laboratory
  - Medical-surgical wards
  - Med-Psychiatric
  - Neuro-stroke
  - Oncology
  - Pediatric
  - Pharmacy
  - Radiology
  - Surgery/OR
  - Other: (please specify) ________________

1. **Which of the following describes your role? (check all that apply)**
   - Physician
   - Advanced Practice Clinician (APC)
   - Leadership/management role
   - Other: (please specify) ________________________
2. **When I am working at the hospital during this time of the COVID-19 pandemic:**

|  | **Strongly disagree** | **Disagree** | **Neutral** | **Agree** | **Strongly agree** |
| --- | --- | --- | --- | --- | --- |
| I feel highly valued. |  |  |  |  |  |
| I feel supported and listened to by my leadership. |  |  |  |  |  |
| I feel comfortable providing feedback or concerns to my leaders. |  |  |  |  |  |
| I believe my concerns will be acted upon. |  |  |  |  |  |

1. **When I am working at the hospital during this time of the COVID-19 pandemic:**

|  | **Strongly disagree** | **Disagree** | **Neutral** | **Agree** | **Strongly agree** | **Not applicable** |
| --- | --- | --- | --- | --- | --- | --- |
| I am worried about my safety at work. |  |  |  |  |  |  |
| My overall well-being has been negatively affected. |  |  |  |  |  |  |
| My childcare or caregiving responsibilities are impacting my work. |  |  |  |  |  |  |
| I am concerned about loss of income. |  |  |  |  |  |  |
| I am concerned about loss of my job. |  |  |  |  |  |  |
| I feel a great deal of stress because of my job. |  |  |  |  |  |  |

1. **What can be done to better support you right now at the hospital? (check all that apply)**
   - More training on COVID-19
   - Provide more personal protective equipment (PPE)
   - More training on use of PPE
   - Support for mental health needs
   - Provide more flexibility with schedules
   - Other: (please specify) ________________________
2. **Please tell us more about what can be done to better support you at the hospital right now?**

(write in response up to 250 words) : ________________________________

Please help us to help you by giving us some background on your role during the COVID-19 pandemic.

1. **How have your responsibilities and work at the hospital changed because of the COVID-19 crisis? (check all that apply)**
   - I have been given additional tasks
   - The hours I work decreased
   - The hours I work increased
   - I voluntarily removed myself from the schedule
   - I began working remotely
   - Nothing changed
   - Other: (please specify) ________________________

Understanding burnout remains an organizational priority, even more so during these difficult times. Please take a moment to reflect on how you are feeling overall about your work.

1. **Overall, based on your definition of burnout, how would you rate your level of burnout?** (*Check only one*)
   - I enjoy my work. I have no symptoms of burnout.
   - Occasionally I am under stress, and I don’t always have as much energy as I once did, but I don’t feel burned out.
   - I am definitely burning out and have one or more symptoms of burnout, such as physical or emotional exhaustion.
   - The symptoms of burnout that I’m experiencing won’t go away. I think about frustration at work a lot.
   - I feel completely burned out and often wonder if I can go on. I am at the point where I may need some changes or may need to seek some sort of help.

**Thank you very much for completing this survey.**

We also encourage physicians and advance practice clinicians (APCs) to seek support when experiencing challenges that are affecting them, a colleague, or family member. It all starts with a conversation:

- **Sutter Health’s Employee Assistance Program (EAP)**—provides free, confidential assistance, including referrals to licensed counseling professionals. Call 1-800-477-2258 or go online to [sutterhealth.org/eap](https://www.sutterhealth.org/for-employees/employee-assistance-program).
- **Physician Support Line** — is a national, free, and confidential support line service made up of 600+ volunteer psychiatrists, joined together in the determined hope to provide peer support for our physician colleagues as we all navigate the COVID-19 pandemic.
  - 1 (888) 409-0141
  - Helping our colleagues all over the U.S. on the front lines of COVID-19
  - Free & Confidential | No appointment necessary
  - Open 7 days a week | 8:00AM - 12:00AM EST
- **National Suicide Prevention Lifeline**—if you are concerned for yourself or someone else, call 1-800-273-8255 .

**Crisis Text Line**—connect by text with a trained crisis counselor by texting 741741.

# Appendix 2. Codebook definition for open-ended comments

| **Major Themes** | **Definitions** |
| --- | --- |
| **Personal protective equipment (PPE) or other equipment/facility needs** | Comments on PPE such as a desire for more PPE/N95, communication about PPE/N95 use, and N95 model options. Any mention of availability and quality of facilities including imaging, labs sites, and operating room. Also includes issues of inefficient use of facilities and space, including availability of beds. Desire for more work supplies on-site (e.g., laptops in respiratory care clinic, internet connection, surgical instruments) and for remote work (e.g., router, laptop, iPad, desktop camera, home office set-up). |
| **Communication with leadership** | Respondent expressed difficulties with communicating to or being heard by leadership. Also included comments around receiving too little or too much information from leadership and wanting better quality of communication (e.g., clearly communicating policies, expectations, etc). For example, respondents commented on a desire to be continually informed, receiving messages viewed as unimportant, contradicting information, and wanting “version control” on communication. Also include requests for financial transparency and communication on financial/income impact or “the plan" for dealing with personal financial losses. |
| **Compensation** | Comments about compensation to clinicians for work including a desire for income fairness between specialties, primary care vs specialists, in-person vs remotely-working clinicians, and within/between departments during this COVID-19 period. Also includes concerns with a productivity pay model, pay cuts for those working in clinic vs remotely, and compensation for hazard pay, leadership obligations, phone and video visits, and benefits such as paid time off. |
| **Working relationships** | Comments seeking improved relationships, collegiality, communication and trust between colleagues, clinicians, and the organization or medical group. This included a desire for improved coordination between different roles (e.g., physicians, nurses, social workers), departments, or larger organization entities. Also included issues with leadership decision-making, and management abilities at any level, lack of on-site presence of leaders, negative work environment, and lack of organizational support to medical group, hospital, and independent physicians. |
| **Positive comments** | Any mention of things going well or have been helpful. Coded by itself for any positive comment. For example, "I feel valued by my local leadership.” |
| **COVID-19 specific training/guidelines** | Requests for the creation and enforcement of clear COVID-specific guidelines relating to: in-person visits, visitation policies, assessment and treatment plans for COVID-19 patients, and protocol regarding access to facilities. |
| **Non-physician, ancillary, & support staff issues** | Comments include requests for more support staff (nurses, medical assistants, physician assistants, LVNs) for video visits or on site. Also include a desire for improved skills and efficiency of support staff working remotely and/or in person. Included any mention of "support staff” or ancillary staff, including requests for more general support for support staff. |
| **COVID-19 Screening** | Desire for Covid-19 testing/screening (including temperature checks) for patient and/or health care professionals because of infection risk concerns. |
| **In-person visits/On-site safety issues** | Any comments on personal health or general safety concerns on site or during in-person visits. Comments on personal health include the ability to hydrate and take breaks. On-site concerns include additional sanitization/cleaning of facilities, desire for personal workstations, hand sanitizers, HEPA filters, and facility security issues. Not coded for comments on PPE/N95. |
| **De-escalation/re-escalation plans** | Comments about decreased patient volumes (pushed from the organization’s side because of COVID-19 closures), not having enough work, timelines to adjust to new work flows (changing too fast, needing more time to ramp up), wanting to return to normal functioning, changes happening too aggressively, expressing desires not to re-escalate and receive increased workload. |
| **Flexibility/Autonomy with schedule** | Wanting flexibility with own schedule to see patients (i.e., who gets scheduled in-person vs. virtual, timing of day for visits), desire to eliminate or relax "Open Encounter" policy (want to be able to add own slots to schedule rather than having to process through other people). Also includes desire to control own administrative time, leadership time, conduct meetings in person or virtually, and have flexibility with time off. |
| **Video visit issues** | Issues and requests relating to video visits or video visit software (i.e., Canto), including appropriateness of video visits for certain departments or patient populations, as well as the view that the potential long-term use of video visits needs to consider the patients that have difficulty utilizing them. Also includes concerns with video visit user experience, visit length, training, and requests for alternative platforms (e.g., Doximity). |
| **Recognition/Appreciation/Positive reinforcement** | Desire to feel valued or recognized for work. Also, wanting improved morale and acknowledgement of individuals, certain positions, or departments. |
| **Work-Life balance** | Conflict with balancing work and personal time. This includes requests for additional benefits such as exercise programs and emotional support for physicians, non-physicians/advanced practice clinicians, and support staff. |
